# Multi-omics integration of molecular genetics, cytogenetics and immunophenotyping: a novel prognostic model for immune landscape characterization and outcome prediction in Chinese patients with acute myeloid leukemia

**DOI:** 10.3389/fimmu.2026.1812970

**Published:** 2026-05-01

**Authors:** Fengli Li, Yangyang Ding, Shanglong Feng, Yingzhao Jin, Beibei Xie, Qing Zhang, Xunyi Jiao, Jinli Zhu, Wanqiu Zhang, Qianshan Tao, Huiping Wang, Depei Wu, Xin Liu, Zhimin Zhai

**Affiliations:** 1 Department of Hematology/Hematological Lab, The Second Hospital of Anhui Medical University, Hefei, China; 2 Department of Hematology, Affiliated Hospital of Jiangnan University, Wuxi, China; 3 Department of Hematology, The First Affiliated Hospital of Soochow University, Suzhou, China; 4 Department of Medicine and Therapeutics, The Chinese University of Hong Kong, Hong Kong, Hong Kong SAR, China; 5 Department of Hematology, The First Affiliated Hospital of USTC, Division of Life Sciences and Medicine, University of Science and Technology of China, Hefei, China

**Keywords:** acute myeloid leukemia, age, CD15, del(7q), genetic risk stratification, hematopoietic stem cell transplantation, multi-omics, prognostic model

## Abstract

**Background:**

Current genetic risk stratification systems for acute myeloid leukemia (AML), including the 2017 European Leukemia Network (ELN), 2022 ELN, and 2023 China (CN) stratifications, inconsistently categorize key genetic variants such as *CEBPA* and *FLT3-ITD*. These systems also demonstrate limited applicability to Chinese patients, complicating clinical decision-making. This study optimized genetic stratification for Chinese AML patients and developed a multi-omics prognostic model by integrating immunophenotypic and clinical characteristics to enhance outcome prediction.

**Methods:**

We retrospectively enrolled 378 Chinese AML patients (298 for training, 80 for external validation) and compared the prognostic performance of the 2017/2022 ELN and 2023 CN systems (both integrating molecular genomic and cytogenetic data). Controversial genetic variants were re-evaluated to establish a novel 2023 CN (n-2023 CN) classification. A multi-omics model was constructed by combining n-2023 CN classification with CD15 positivity (leukemic immunophenotype), del(7q) (cytogenetic abnormality), and age ≥ 60 years (clinical factor). The model underwent external validation, was assessed for use in guiding hematopoietic stem cell transplantation (HSCT), and was compared with a published model.

**Results:**

The 2023 CN demonstrated superior efficacy in predicting AML relapse and 3-year relapse-free survival compared to 2017/2022 ELN systems. The n-2023 CN reclassified *CEBPA*^bZIP^ as favorable-risk, t(8;21)/inv(16) with *KIT*^D816^/*FLT3-ITD*^low^ as intermediate-risk, and *FLT3-ITD*^high^ as adverse-risk among patients not receiving *FLT3* inhibitors (regardless of *NPM1* co-mutation). CD15 positivity, del(7q), and age ≥ 60 years were independent adverse prognostic factors for overall survival (OS). The model stratified patients into three risk groups with significantly distinct complete remission (CR) rates (93.2%, 71.2%, 35.7%) and OS (*P* < 0.001), with a concordance index of 0.729 (internal) and 3-year OS AUC of 0.752 (external). High-risk patients receiving HSCT exhibited longer OS. The model outperformed a published comparator in predicting CR and 1- and 3-year OS (all *P* ≤ 0.001).

**Conclusions:**

The n-2023 CN resolves uncertainty in genetic risk stratification for Chinese AML patients. The novel multi-omics model incorporates immunophenomics and clinical and cytogenetic factors, captures comprehensive AML characteristics, enhances prognostic precision, and provides reliable guidance for personalized treatment, including HSCT selection.

## 1 Introduction

Acute myeloid leukemia (AML) is a genetically heterogeneous hematological malignancy characterized by clonal expansion of immature myeloid progenitors and highly variable clinical outcomes (1). Accurate risk stratification is the cornerstone of personalized therapeutic decision-making for AML and is closely associated with patients’ long-term survival.

Current clinical guidelines, including the 2017/2022 European LeukemiaNet (ELN) and 2023 Chinese (CN) systems, demonstrate inconsistent classification of specific genetic variants (2–4). For instance, prognostic definitions diverge significantly for *CEBPA* mutation (biallelic versus bZIP in-frame status) and core-binding factor leukemia (CBF-AML) harboring the *KIT*^D816^ mutation. Similarly, the risk assignment for *FLT3-ITD*, particularly regarding the allelic ratio and concurrent *NPM1* mutation, as well as myelodysplasia-related (MR) gene mutation and *TP53* variant allele fraction (VAF), remains controversial across these systems. These classification discrepancies, combined with potential ethnic genetic variations, necessitate comprehensive validation of these systems in Chinese AML patients (5–11). More importantly, these systems fail to incorporate clinical and immunophenotypic factors with potential prognostic value, leading to unresolved prognostic heterogeneity within the same risk group.

Leukemic cell immunophenotypic characteristics serve as core diagnostic criteria and emerging prognostic biomarkers that complement genetic stratification. Specific cell surface markers of leukemic blasts have been reported to correlate with tumor biological behavior and treatment response in AML. CD15 positivity is an independent adverse prognostic marker associated with cutaneous infiltration, Bcl-2 overexpression, and *DNMT3A/FLT3-ITD* mutations, all of which contribute to chemotherapy resistance and inferior survival (12–15). Immunophenotypic features, including positivity for CD7, CD34, CD44, CD56, and CD117, are similarly associated with lower remission rates, elevated relapse risk, and dismal clinical outcomes (16–20). In contrast, expression of CD11b, CD19, CD33, CD38, and MPO predicts a more favorable prognosis (21–24). Furthermore, clinical factors reflecting patient physiological status, along with certain under-evaluated cytogenetic abnormalities, heavily influence treatment tolerance and are closely associated with poor long-term survival outcomes in Chinese AML patients (25). Elderly patients with AML frequently harbor myelodysplasia-related gene mutations (including *SF3B1* and *SRSF2*), *TP53* mutation, and complex karyotypes (11). They often present with multiple comorbidities, poor treatment tolerance, and high early mortality. Multiple studies have demonstrated that age ≥ 60 years is an independent adverse prognostic factor in AML (26–28). Collectively, these unintegrated factors may carry independent prognostic value and deserve further evaluation. With the advancement of high-throughput detection technologies, multi-omics integration has become a feasible strategy to dissect tumor heterogeneity and improve prognostic assessment precision in oncology; by combining genetic, immunophenotypic, and clinical data, prognostic models can capture multi-dimensional biological characteristics and overcome the limitations of traditional dual-omics stratification, though relevant studies in Chinese AML populations are still limited.

In this study, we enrolled a cohort of Chinese AML patients, all of whom were treated with intensive chemotherapy. We first re-evaluated the prognostic value of controversial genetic variants and optimized the existing genetic stratification to establish the novel 2023 China (n-2023 CN) classification, and then constructed a multi-omics prognostic model by integrating the n-2023 CN stratification with specific immunophenotypic, cytogenetic, and clinical factors identified in our analysis. We aimed to investigate the prognostic performance of this integrated model and its value in guiding HSCT, to provide a more refined risk stratification tool for Chinese AML patients.

## 2 Methods

### 2.1 Patients and treatment

A total of 378 patients newly diagnosed with acute myeloid leukemia (excluding acute promyelocytic leukemia [APL]) were enrolled from three medical centers between 2015 and 2023. All patients received intensive induction chemotherapy based on a “3 + 7” IA/DA regimen. Patients were assigned to a training cohort (n = 298) and an external validation cohort (n = 80). This study was approved by the Institutional Review Board and conducted in accordance with the Declaration of Helsinki.

### 2.2 Multi−omics profiling

Multi-omics data, including next-generation sequencing, cytogenetics, and immunophenotyping, were obtained from diagnostic bone marrow or peripheral blood samples.

### 2.3 Definitions and statistical analysis

The primary endpoints were overall survival (OS) and relapse−free survival (RFS). Model construction and validation were performed using Cox regression, LASSO−Cox regression, ROC curves, calibration curves, and decision curve analysis. All statistical analyses were performed using R software (version 4.2.0).

Detailed experimental protocols, patient eligibility, and statistical procedures are provided in the **Supplementary Materials**.

## 3 Results

### 3.1 Baseline characteristics

**Figure 1:**
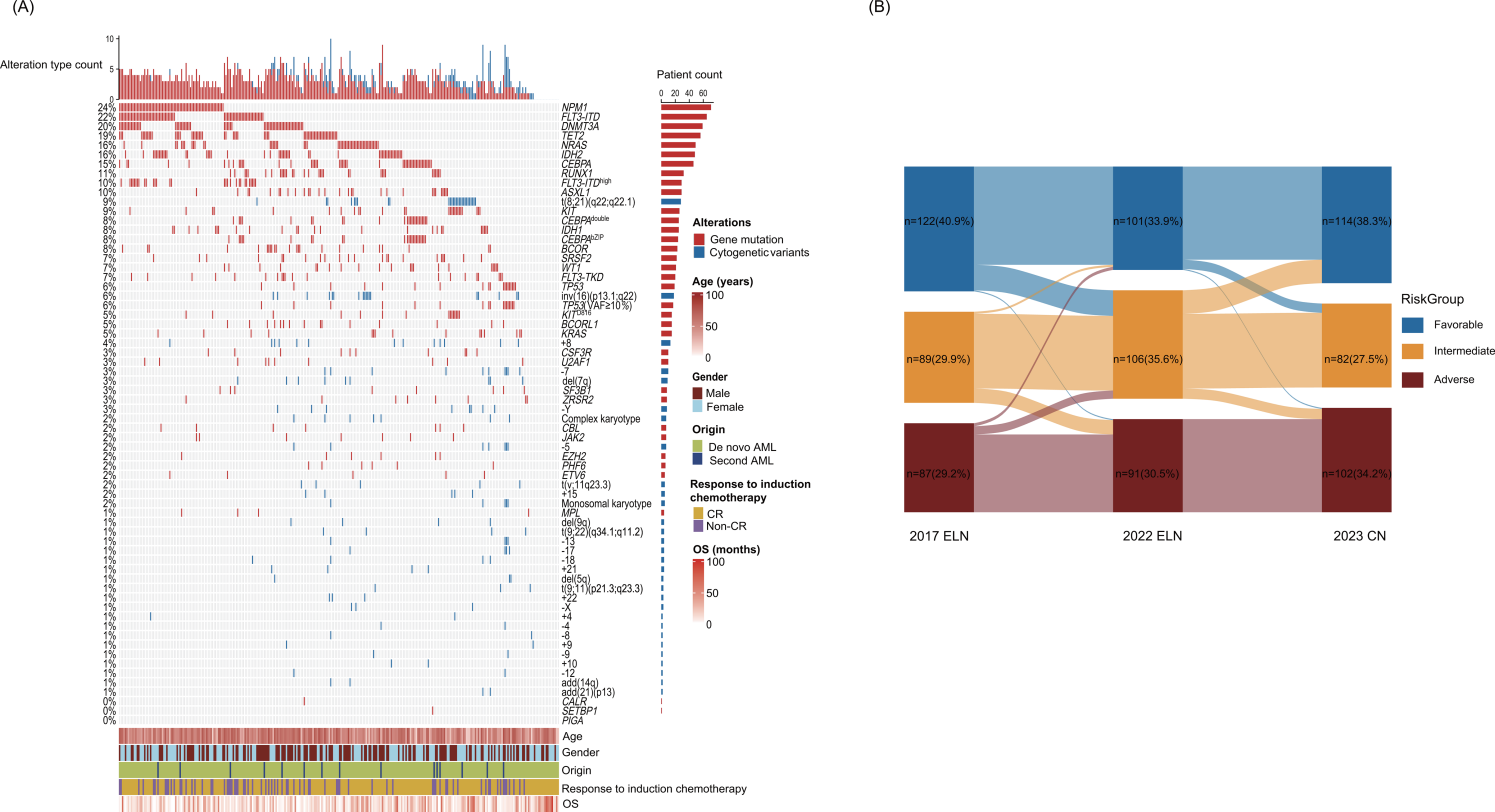
Patient baseline characteristics and distributions by the 2017, 2022 ELN, and 2023 CN. **(A)** Waterfall and **(B)** alluvial diagram. *CEBPA*^biallelic^, biallelic *CEBPA* mutation; VAF, variant allele fraction.

This training cohort included 298 patients comprising 283 individuals with *de novo* AML and 15 with secondary AML (S-AML). The median age of the patient cohort was 54.5 years (range 15-81), and 48.7% were male. After 1-2 induction chemotherapy cycles, 71.5% achieved CR, with 49.3% relapsing. HSCT was conducted in 66 patients, usually post-induction or at relapse, and 67 patients received venetoclax. Our cohort had a median follow-up of 42.4 months and a median OS of 17.8 months (range, 0.1-94.8 months). Among the 213 patients who achieved CR after induction chemotherapy, the median RFS was 15 months (range, 0.5-93.6 months). **Table 1** and **Figure 1A** detail patient demographics and the relationships among gene mutations, chromosomal aberrations, survival outcomes, and clinical features.

**Table 1:** Baseline characteristics of patients.

Characteristics	Values
Age, years	54.5 (15-81)
< 60	189 (63.4%)
≥ 60	109 (36.6%)
Sex (male/female)	145/153
BM blasts, %	58.5 (3.5-97)
WBC, 10^9^/L	14.3 (0.4-440)
HB, g/L	75 (32-138)
PLT, 10^9^/L	40 (3-485)
FAB-classification	
M0	2 (0.7%)
M1	17 (5.7%)
M2	117 (39.3%)
M4	38 (12.8%)
M5	66 (22.1%)
M6	1 (0.3%)
Not available	57 (19.1%)
Origin of disease	
*De novo* AML	283 (95%)
S-AML	15 (5%)
2017 ELN	
Favorable	122 (40.9%)
Intermediate	89 (29.9%)
Adverse	87 (29.2%)
2022 ELN	
Favorable	101 (33.9%)
Intermediate	106 (35.6%)
Adverse	91 (30.5%)
2023 CN	
Favorable	114 (38.3%)
Intermediate	82 (27.5%)
Adverse	102 (34.2%)
Treatment	
+ Venetoclax	67 (19.1%)
**CR**	213 (71.5%)
Relapse	105 (49.3%)
OS, months	17.8 (0.1-94.8)
RFS, months	15 (0.5-93.6)

BM, bone marrow; WBC, white blood cell; HB, hemoglobin; PLT, platelet; FAB, French-American-British; S-AML, secondary AML; ELN, European Leukemia Net; CN, China; CR, complete remission; OS, overall survival; RFS, relapse-free survival.

### 3.2 Comparison and validation of predictive accuracy of the three existing genetic risk stratifications for survival outcomes

**Figure 2:**
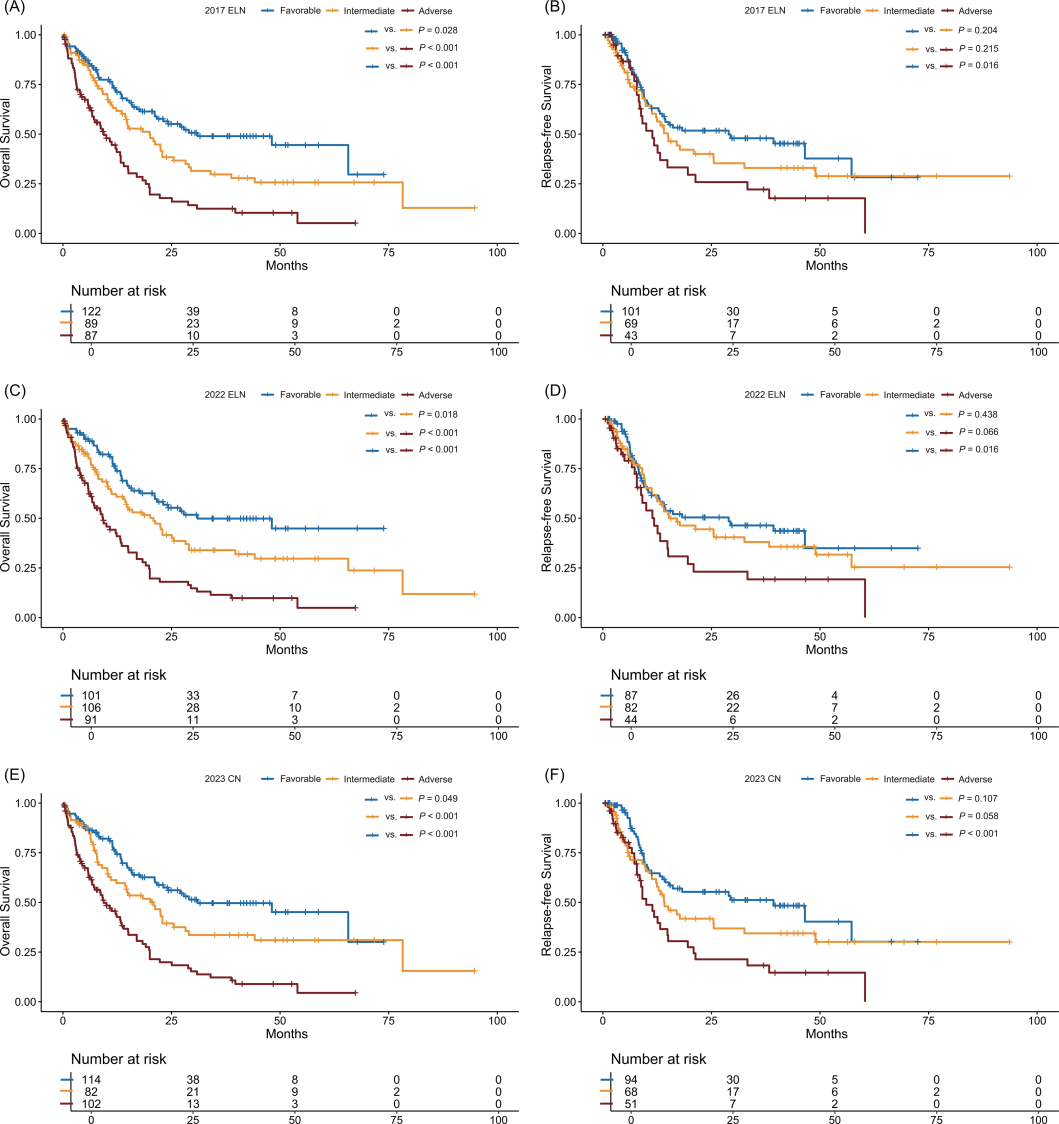
Survival outcomes of patients according to the 2017 ELN, 2022 ELN, and 2023 CN. **(A)** Overall survival and **(B)** relapse-free survival of patients according to the 2017 ELN. **(C)** Overall survival and **(D)** relapse-free survival of patients according to the 2022 ELN. **(E)** Overall survival and **(F)** relapse-free survival of patients according to the 2023 CN.

Under the 2017 ELN, the patients were distributed as follows: favorable (n = 122), intermediate (n = 89), and adverse (n = 87). With the 2022 ELN update, 53 patients were reassigned to different risk groups, resulting in new distributions. Compared to the 2022 ELN, the 2023 CN regrouped 43 patients into favorable- (n = 114), intermediate- (n = 82), and adverse- (n = 102) risk groups. **Figure 1B** (alluvial diagram) illustrates these shifts in genetic risk stratifications and distributional changes, with details in **Supplementary Tables 1**, **S2**. **Supplementary Table 3** presents CR rates, relapse incidence, and 1- and 3-year survival for each genetic risk-stratification group. OS stratification was clear across risk groups in the 2017 ELN, 2022 ELN, and 2023 CN analyses, with significant differences between favorable- and intermediate-risk groups, and between intermediate- and adverse-risk groups (**Figures 2A, C, E**). However, RFS did not show distinct stratification, with no significant differences between favorable- and intermediate-risk groups, intermediate- and adverse-risk groups (**Figures 2B, D, E**).

Using ROC curves, the three genetic risk stratification systems had similar predictive power for CR (AUCs: 0.674 for 2017 ELN, 0.699 for 2022 ELN, 0.679 for 2023 CN; no significant differences: 2017 ELN vs. 2022 ELN, *P* = 0.207; 2017 ELN vs. 2023 CN, *P* = 0.718; 2022 ELN vs. 2023 CN, *P* = 0.245) (**Supplementary Figure 1A**). For relapse prediction, the 2023 CN was more effective (AUCs: 0.57 for 2017 ELN, 0.547 for 2022 ELN, 0.603 for 2023 CN; 2023 CN vs. 2017 ELN, *P* = 0.044; 2023 CN vs. 2022 ELN, *P* = 0.003) (**Supplementary Figure 1B**). For survival prediction, they had similar efficacy for 1-year OS, 3-year OS, and 1-year RFS (*P* > 0.05), but the 2023 CN was better at predicting 3-year RFS than the 2017 ELN (2023 CN vs. 2017 ELN, *P* = 0.006) (**Supplementary Figures 1C–F**).

### 3.3 Assessment of the controversial genetic variants and construction of the new genetic risk stratification

We performed an in-depth analysis of genetic variants with controversial risk classifications across the 2017 ELN, 2022 ELN, and 2023 CN AML risk stratification systems.

#### 3.3.1 *CEBPA* ^bZIP(biallelic)^

Among 46 *CEBPA*-mutated patients, 18 had *CEBPA*^bZIP^ with biallelic mutation (*CEBPA*^biallelic^), 2 had monoallelic *CEBPA*^bZIP^ mutation, 4 had a non-bZIP in-frame biallelic mutation, and 22 had non-bZIP in-frame monoallelic mutations. We separately compared *CEBPA*^bZIP(± biallelic)^ (*CEBPA*^bZIP^ regardless of whether it was also *CEBPA*^biallelic^), *CEBPA*^biallelic(± bZIP)^ (*CEBPA*^biallelic^ regardless of whether it was also *CEBPA*^bZIP^), *CEBPA*^bZIP(only)^ (*CEBPA*^bZIP^ with monoallelic mutation), *CEBPA*^biallelic(only)^ (*CEBPA*^biallelic^ that was not a bZIP in-frame mutation), and the rest favorable (favorable -risk group excluding concomitant genetic variants that could affect the risk, such as *TP53*, *FLT-ITD*^high^, t(8;21)(q22;q22) or inv(16)(p13q22)/t(16;16)(p13;q22)-*KIT*^D816^) for clinical outcomes. The OS of patients in the first three groups was not statistically different from that of the rest, which was favorable. In contrast, the OS of *CEBPA*^biallelic(only)^ was significantly worse than that of the rest of the favorable group (**Supplementary Figures 2A, B**). When focusing on RFS, unfortunately, the two patients with *CEBPA*^bZIP(only)^ failed to achieve CR after induction chemotherapy. However, the RFS of patients with *CEBPA*^biallelic(only)^ was significantly worse than the rest favorable, showing the same trend as OS (**Supplementary Figures 3A, B**). These findings support the inclusion of *CEBPA*^bZIP^ but not *CEBPA*^biallelic^ in the favorable-risk stratification.

#### 3.3.2 t(8;21)(q22;q22) or inv(16)(p13q22)/t(16;16)(p13;q22) with mutated *KIT*^D816^

Our study assessed the influence of t(8;21)(q22;q22) or inv(16)(p13q22)/t(16;16)(p13;q22) (hereafter abbreviated as t(8;21)/inv(16)) and the *KIT*^D816^ mutation on OS and RFS. Patients with t(8;21)/inv(16) without *KIT*^D816^ mutation showed a trend toward better OS than those intermediate without any genetic variants (the 2017 ELN, 2022 ELN, and 2023 CN all classified “genetic variants not classified as favorable or adverse” into the intermediate-risk group, so we defined these as “intermediate without any genetic variants”). However, this did not reach statistical significance (*P* = 0.069, **Supplementary Figure 2C**). Patients with both t(8;21)/inv(16) and *KIT*^D816^ mutation had significantly poorer OS and RFS than those without the *KIT*^D816^ mutation (**Supplementary Figures 2C**, **S3C**). Patients with t(8;21)/inv(16) carrying the *KIT*^D816^ mutation had no significant difference in OS compared with intermediate without any genetic variants (**Supplementary Figure 2C**). However, RFS was worse for patients with the *KIT*^D816^ mutation than for those with intermediate risk without any genetic variants (**Supplementary Figure 3C**), supporting classification of t(8;21)/inv(16) and *KIT*^D816^ as intermediate risk (in the absence of other adverse variants).

#### 3.3.3 *FLT3-ITD*(± *NPM1*)

We analyzed the prognostic impact of *FLT3-ITD* allelic ratios and *NPM1* mutation by excluding patients with concurrent favorable or adverse genetic variants. We compared *FLT3-ITD(± NPM1)* (mutated *FLT3-ITD* regardless of whether it is combined with *NPM1* mutation), *FLT3-ITD*^high^*(± NPM1)*, *FLT3-ITD*^low^*(± NPM1)*, and *FLT3-ITD-NPM1* (mutated *FLT3-ITD* and *NPM1*) groups to intermediate without any genetic variants. Our study showed that when the allelic ratio was not taken into account, *FLT3-ITD*-mutated patients, regardless of *NPM1* status, had inferior CR rate, OS, and RFS compared with intermediate patients without any genetic variants (**Supplementary Table 4**; **Supplementary Figures 2D**, **S3D**). *FLT3-ITD*^high^ patients also had worse OS and RFS than intermediate patients without any genetic variants, irrespective of *NPM1* mutation (**Supplementary Figures 2E, F**, **S3E, F**). *FLT3-ITD*^low^ patients with *NPM1* mutation did not differ significantly in OS and RFS but had a lower CR rate than intermediate patients without any genetic variants (**Supplementary Table 4**).

In the entire cohort, 65 patients had an *FLT3-ITD* mutation: 29 were *FLT3-ITD*^high^, 14 received *FLT3* inhibitors, and 36 were *FLT3-ITD*^low^, of whom 7 received inhibitors. This reflects the limited accessibility of inhibitors in Chinese clinical practice. Excluding the 22 patients with favorable or adverse variants, only 13 out of the remaining 43 received *FLT3* inhibitors. This reflects limited access to inhibitors in Chinese clinical practice; thus, we should focus more on the prognosis of *FLT3-ITD* in the absence of inhibitors.

We compared *FLT3-ITD*^high^*(± NPM1)*, *FLT3-ITD*^low^*(± NPM1)*, *FLT3-ITD*^high^*-NPM1*, and *FLT3-ITD*^low^-*NPM1* groups, with and without inhibitors, to the intermediate group without any genetic variants (**Supplementary Figure 4**). Our results showed that, in the absence of inhibitors, patients had worse OS than intermediate without any genetic variants, regardless of the presence of a concurrent *NPM1* mutation and regardless of the high or low allele ratio of *FLT3-ITD*. In contrast, in patients receiving inhibitors, OS did not differ from that of intermediate without any genetic variants. However, when RFS was analyzed, the opposite result was observed: patients using inhibitors had a significantly shorter RFS than intermediate patients without any genetic variants. The CR rate in the *(NPM1-)FLT3-ITD* inhibitor group was 100%. Only four patients started inhibitor therapy during induction, each achieving CR. The remaining patients began inhibitor use post-CR or upon relapse (9 out of 13, 69.2%). Consequently, the above prolongation of OS in the inhibitor-using group may have been attributable more to CR achieved through intensive induction chemotherapy than to the inhibitor itself. These outcomes support classifying *FLT3-ITD*^high^ as adverse risk and *FLT3-ITD*^low^ as intermediate risk (regardless of *NPM1*) in the absence of inhibitors.

#### 3.3.4 MR genes

Compared with MR gene carriers without favorable variants in either the 2022 ELN or the 2023 CN, those with favorable variants had higher CR rates, OS, and RFS (**Supplementary Table 4**; **Supplementary Figures 2G**, **S3G**). Furthermore, when evaluating clinical outcomes of MR genes without additional variants (compared to intermediate without any genetic variants), MR genes without additional variants showed inferior outcomes in CR rate, OS, and RFS (**Supplementary Table 4**; **Supplementary Figures 2H**, **S3H**). Therefore, our findings support this adjustment in the 2022 ELN and the 2023 CN.

#### 3.3.5 *TP53* (VAF ≥ 10%)

Our study assessed the impact of *TP53* VAF on CR, relapse, OS, and RFS but found no significant differences due to the limited sample size (**Supplementary Table 4**; **Supplementary Figures 2I**, **S3I**).

**Figure 3:**
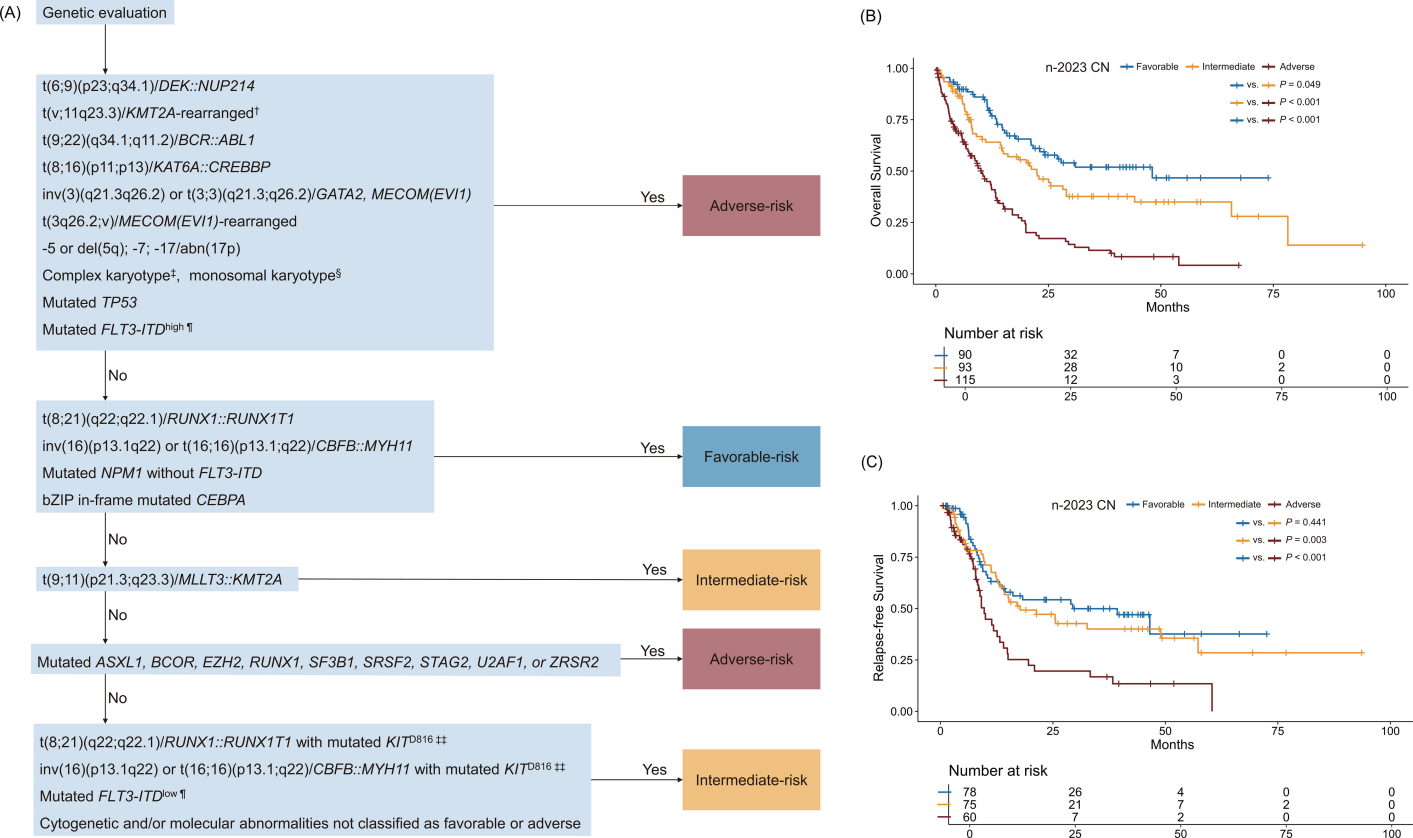
Workflow and prognostic performance of the n−2023 CN. †, Excluding *KMT2A* partial tandem duplication; ‡, ≥ 3 unrelated chromosome abnormalities without other class-defining recurring genetic abnormalities; excludes hyperdiploid karyotypes with three or more trisomies (or polysomies) without structural abnormalities; §, Presence of two or more distinct monosomies (excluding loss of X or Y) or one single autosomal monosomy in combination with at least one structural chromosome abnormality (excluding core-binding factor AML); ¶, Low, low allelic ratio (< 0.5); high, high allelic ratio (≥ 0.5). ‡‡, *KIT*^D816^, the D816 locus of *KIT*.

Based on the above analysis, we adjusted the classification of five key genetic variants in the 2023 CN, thereby forming the n-2023 CN stratification system, as shown in the simplified flowchart in **Figure 3A**. Compared with the 2017 ELN, 2022 ELN, and 2023 CN, the n- 2023 CN demonstrated enhanced discriminatory power, precisely stratifying patients into favorable (n = 90), intermediate (n = 93), and adverse-risk (n = 115) groups. The n-2023 CN favorable group had a notably high CR rate of 86.7%, exceeding the CR rates of favorable groups in the other systems. This indicates its ability to identify patients who are more likely to respond well to treatment. The relapse rates in the n-2023 CN defined groups also showed a more distinct separation. For example, the adverse group had a relapse rate of 60%, clearly highlighting the higher-risk patients. For survival, the median OS for the favorable group was 48.1 months, far exceeding the corresponding values in the other systems, and the difference in median OS between the intermediate and adverse groups was more pronounced, with *P* values (favorable-intermediate, *P* = 0.049; intermediate-adverse, *P* < 0.001) demonstrating greater statistical significance (**Supplementary Table 5**; **Figure 3B**). Similarly, RFS stratification between the intermediate and adverse groups was significant (*P* = 0.003; **Figure 3C**). DeLong’s test showed that the n-2023 CN demonstrated a significantly higher AUC for predicting 3-year OS (AUC = 0.68) compared to the 2017 ELN (AUC = 0.636, *P* = 0.012) and 2023 CN (AUC = 0.648, *P* = 0.006). For predicting 1-year RFS, the n-2023 CN’s AUC (0.588) was statistically superior to the 2022 ELN (AUC = 0.546, *P* = 0.03). In predicting 3-year RFS, the n-2023 CN further exhibited a significantly elevated AUC (0.616) compared to both the 2017 ELN (AUC = 0.562, *P* = 0.005) and 2022 ELN (AUC = 0.574, *P* = 0.007; **Supplementary Figure 5**).

### 3.4 Construction and risk stratification performance of the multi−omics prognostic model integrating molecular genomics, cytogenomics and immunophenomics

We further explored the prognostic impacts of a comprehensive set of clinical and biological factors on OS. In the univariate Cox regression analysis, we incorporated the newly established n-2023 CN alongside a wide range of baseline variables. These variables included clinical features (age, origin of disease, sex, and venetoclax treatment history), laboratory parameters and immunophenotypic markers (WBC, BM blasts, hyperleukocytic leukemia status, PLT, HB, CD15, CD19, CD2, CD4, CD7, CD11b, CD11c, CD13, CD14, CD22, CD33, CD34, CD38, CD45, CD56, CD64, CD71, CD79a, CD117, and HLA-DR), genetic factors (total count of gene mutations, *DNMT3A*, *IDH2*, *TET2*, *BCORL1*, *CBL*, *CSF3R*, *ETV6*, *FLT3-TKD*, *IDH1*, *JAK2*, *KRAS*, *NRAS*, *PHF6*, *WT1*), as well as specific chromosomal karyotypes (del(7q), +8, -Y, and +15), all of which are independent of the parameters used within the n-2023 CN stratification.

Univariate Cox regression identified 11 factors significantly associated with OS (*P* < 0.05): age, WBC count, origin of disease, gene mutations count, *DNMT3A*, *IDH2*, *TET2*, CD15, CD19, del(7q), and n-2023 CN (**Table 2**). To ensure the validity of this variable selection process and to assess potential multicollinearity among these 11 candidate variables, we performed a VIF analysis. VIF analysis confirmed the absence of multicollinearity among these variables (VIF range: 1.09-1.36, all <5) (**Supplementary Table 6**). Subsequently, these 11 significant variables were incorporated into a multivariable Cox proportional hazards regression model. Multivariable Cox regression identified four independent adverse prognostic factors for OS: age ≥ 60 years (HR = 1.941, 95% CI 1.396-2.697, *P* < 0.001), CD15 positivity (HR = 1.579, 95% CI 1.117-2.23, *P* = 0.01), del(7q) (HR = 3.83, 95% CI 1.671-8.777, *P* = 0.002), and adverse n-2023 CN classification (HR = 2.738, 95% CI 1.751-4.282, *P* < 0.001) (**Table 2**).

**Table 2:** Univariate and multivariate analysis for survival of patients.

Variables	Comparison	Cox univariate analysis	Cox multivariate regression		
		*P* value	HR (95% CI)	Regression coefficients (β)	*P* value
**Age, years**	≥ 60 vs. < 60	**< 0.001**	1.94 (1.396-2.697)	0.663	**< 0.001**
**WBC, 10^9^/L**	≥ 25 vs. < 25	**0.013**	1.237 (0.881-1.738)	0.213	0.219
**Origin of disease**	S-AML vs. *de novo* AML	**0.011**	1.38 (0.713-2.672)	0.322	0.339
**Gene mutations count**	≥ 4 vs. < 4	**< 0.001**	1.007 (0.666-1.52)	0.006	0.975
***DNMT3A***		**0.021**	1.04 (0.699-1.546)	0.039	0.848
***IDH2***		**0.031**	1.33 (0.883-2.004)	0.285	0.172
***TET2***		**0.008**	1.374 (0.921-2.049)	0.318	0.119
**CD15**		**0.035**	1.579 (1.117-2.23)	0.457	**0.01**
**CD19**		**0.021**	0.948 (0.463-1.943)	-0.053	0.885
**del(7q)**		**< 0.001**	3.83 (1.671-8.777)	1.343	**0.002**
**n-2023 CN**	Adv vs. Fav	**< 0.001**	2.738 (1.751-4.282)	1.007	**< 0.001**
	Int vs. Fav	**0.049**	1.371 (0.871-2.159)	0.315	0.173
Sex	Male vs. female	0.236			
BM blasts, %	≥ 50 vs. < 50	0.263			
Hyperleukocytic leukemia		0.199			
PLT, 10^9^/L	< 69 vs. ≥ 69	0.186			
HB, g/L	< 52 vs. ≥ 52	0.083			
Treat with venetoclax	not vs. yes	0.235			
*BCORL1*		0.053			
*CBL*		0.963			
*CSF3R*		0.124			
*ETV6*		0.853			
*FLT3-TKD*		0.225			
*IDH1*		0.499			
*JAK2*		0.339			
*KRAS*		0.107			
*NRAS*		0.383			
*PHF6*		0.723			
*WT1*		0.761			
CD2		0.813			
CD4		0.079			
CD7		0.319			
CD11b		0.052			
CD11c		0.429			
CD13		0.859			
CD14		0.396			
CD22		0.333			
CD33		0.721			
CD34		0.515			
CD38		0.546			
CD45		0.449			
CD56		0.479			
CD64		0.438			
CD71		0.197			
CD79a		0.547			
CD117		0.721			
HLA-DR		0.768			
+8		0.066			
-Y		0.848			
+15		0.201			

χ2, Chi-Square test; HR, hazard ratio; CI, confidence interval; vs. versus; WBC, white blood cell; S-AML, secondary AML; BM, bone marrow; PLT, platelet; HB, hemoglobin; Adv, Adverse; Fav, Favorable; Int, Intermediate. Footnote: Bold values denote statistically significant differences between groups (*P* < 0.05).

**Figure 4:**
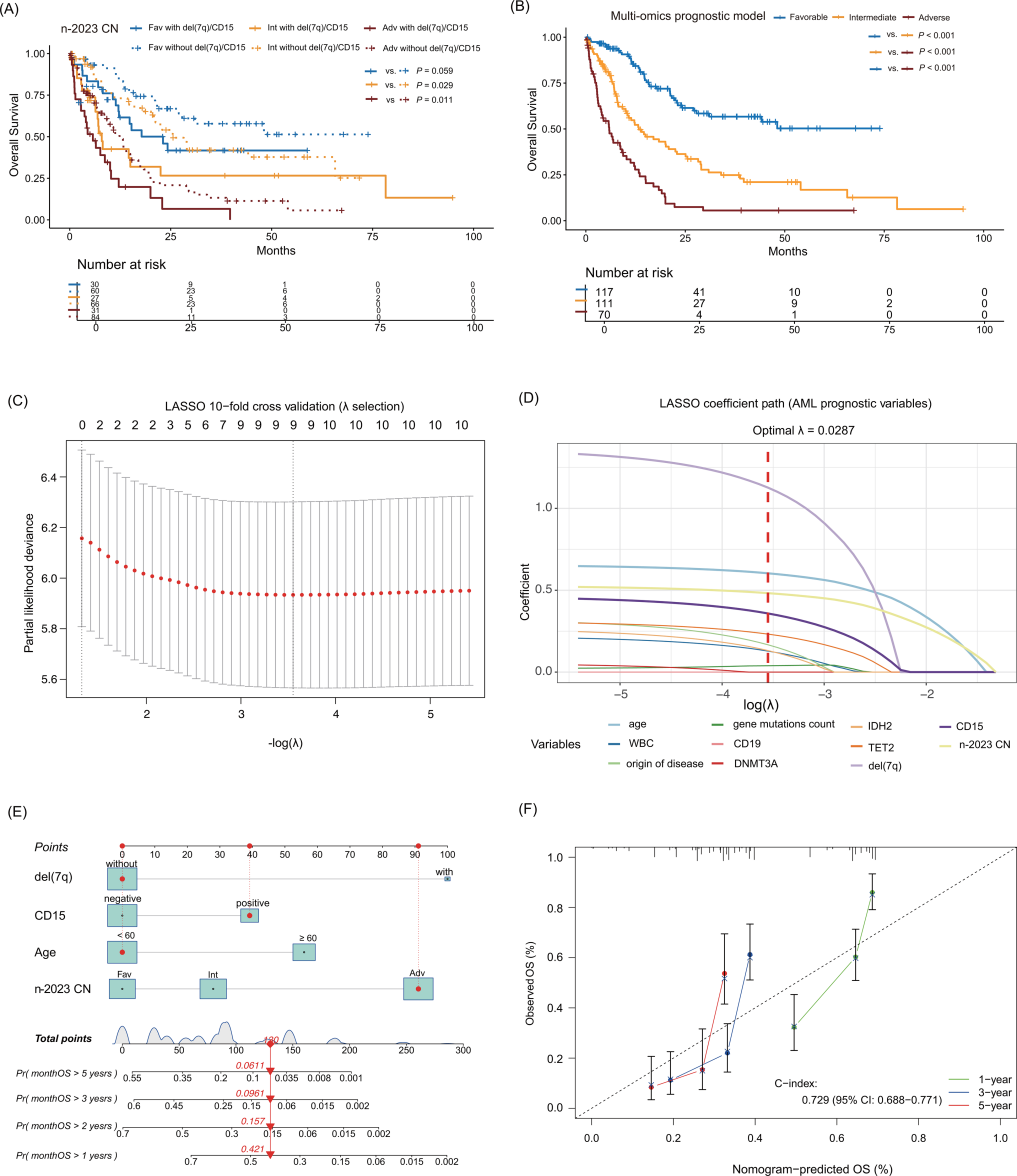
Variable selection, construction, and internal validation of the multi−omics prognostic model. **(A)** Overall survival of patients with/without del(7q)/CD15 according to the n−2023 CN. **(B)** Overall survival of patients according to the multi−omics prognostic model. **(C)** LASSO 10−fold cross validation for λ selection. **(D)** LASSO coefficient path for AML prognostic variables. **(E)** Nomogram constructed based on the multi−omics prognostic model. **(F)** Calibration curve of the nomogram for internal validation. Fav, Favorable; Fav with del(7q)/CD15, Favorable-risk patients with del(7q) and/or CD15 positivity; Fav without del(7q)/CD15, Favorable-risk patients without del(7q) or CD15 positivity; Int, Intermediate; Int with del(7q)/CD15, Intermediate-risk patients with del(7q) and/or CD15 positivity; Int without del(7q)/CD15, Intermediate-risk patients without del(7q) or CD15 positivity; Adv, Adverse; Adv with del(7q)/CD15, Adverse-risk patients with del(7q) and/or CD15 positivity; Adv without del(7q)/CD15, Adverse-risk patients without del(7q) or CD15 positivity; λ, penalty coefficient; WBC, white blood cell; CN, China.

Notably, we further evaluated the prognostic impact of CD15 positivity and del(7q) within each n-2023 CN risk subgroup using Kaplan-Meier survival analysis. Kaplan-Meier subgroup analysis confirmed CD15 positivity and del(7q) predicted inferior OS across all n-2023 CN risk categories: favorable-risk group (*P* = 0.059), intermediate-risk group (*P* = 0.029), adverse-risk group (*P* = 0.011) (**Figure 4A**).

To explore the biological underpinnings of CD15’s adverse prognostic effect, we first characterized the clinical and biological disparities between CD15-positive and CD15-negative patients in the training cohort (**Supplementary Table 7**). No significant differences were observed in age, gender distribution, hemoglobin level, platelet count, or disease origin (*de novo* vs. secondary AML) between the two groups (all *P* > 0.05). However, CD15-positive patients exhibited higher bone marrow blast percentage (69.4% vs 53.3%, *P* = 0.002), peripheral WBC count (25.6 vs 10.6×10^9^/L, *P* = 0.002), M5 FAB subtype predominance (47.8% vs 19.2%, *P* < 0.001), post-remission relapse rate (65.5% vs 49.4%, *P* = 0.04), and extramedullary disease incidence (17.5% vs 6.9%, *P* = 0.018) (**Supplementary Table 7**). We next investigated the correlation between CD15 expression levels and peripheral blood lymphocyte subsets in 57 patients with complete immunophenotypic data for lymphocyte subpopulations (**Supplementary Figure 6**). Spearman correlation analysis revealed that CD15 expression was significantly and negatively correlated with the proportion of NK cells among peripheral blood nucleated cells (Spearman r = -0.380, *P* = 0.004) and showed a borderline significant negative correlation with the percentage of NK cells relative to total lymphocytes (Spearman r = -0.304, *P* = 0.022). No other notable correlations were identified between CD15 expression and the distribution of peripheral blood lymphocyte subsets (all *P* > 0.05).

Based on these robust, statistically validated findings, we constructed a final multi-omics prognostic model incorporating these four variables. The patient risk score was formulated using the respective regression coefficients (β) derived from the multivariate Cox model (**Table 2**). Specifically, the risk score was calculated as follows: 0.663 × age (1 for ≥ 60 years; 0 for < 60 years) + 0.457 × CD15 (1 for positive; 0 for negative) + 1.343 × del(7q) (1 for present; 0 for absent) + the n-2023 CN status (1.007 for adverse; 0.315 for intermediate; 0 for favorable). By determining the cumulative risk score for each individual, the cohort was stratified into three distinct prognostic groups: favorable-risk (score [0, 0.77)), intermediate-risk (score [0.77, 1.66)), and adverse-risk (score ≥ 1.66).

This multi-omics prognostic model stratified patients into favorable- (n = 117), intermediate- (n = 111), and adverse-risk (n = 70) groups, with CR rates of 93.2%, 71.2%, and 35.7% (all pairwise *P* < 0.001) and relapse rates of 38.5%, 59.5%, and 64% (**Supplementary Table 5**). Median OS significantly differed between all risk categories (all *P* < 0.001) (**Figure 4B**). The multi-omics prognostic model outperformed these four systems in predicting CR, relapse, 1/3-year OS, and 1-year RFS (all *P* < 0.05) (**Supplementary Figure 5**).

Additionally, to verify the robustness of the multivariable Cox proportional hazards regression model that incorporates four core variables, including age, del(7q), CD15, and n−2023 CN, we performed LASSO-penalized Cox regression analysis in the training cohort (n = 298). A total of 11 candidate predictors with significant prognostic value identified in the univariate analysis were included in the LASSO model. As shown in **Figure 4C**, we used 10-fold cross-validation to select the optimal penalty parameter λ. The optimal value was determined as λ.min = 0.0287, which yielded the minimum partial likelihood deviance and was chosen to balance model complexity and predictive accuracy.

Under this optimal λ value, **Figure 4D** illustrates the LASSO coefficient path: the LASSO-Cox model ultimately retained nine predictors with non-zero coefficients, all of which included the four core variables (age, del(7q), CD15, and n−2023 CN). Notably, del(7q), age, n−2023 CN, and CD15 exhibited the largest and most persistent positive coefficients across a wide range of λ values, which indicates their strong and stable contributions to adverse prognosis. In contrast, *TET2*, origin of disease, *IDH2*, WBC count, and gene mutations count showed only moderate coefficients and narrow non-zero intervals, suggesting that their prognostic information is relatively weak or partially overlaps with that of the aforementioned core variables. *DNMT3A* and CD19 had coefficients consistently close to 0 across most penalty intensities, implying limited independent predictive value once key variables are already included. Collectively, these findings confirm that the four variables selected in the final Cox model are the most robust and informative prognostic factors in our cohort.

Based on this prognostic model, a nomogram was constructed to predict 1-, 2-, and 3-year OS. The calibration curve analysis and the concordance index (C-index), derived from 1,000 bootstrap resampling iterations (0.729; 95% confidence interval: 0.688-0.771), confirmed the predictive utility of this nomogram (**Figures 4E, F**).

### 3.5 External validation and clinical translation of the multi−omics prognostic model

An independent external validation cohort of 80 patients from another hospital was established, comprising patients who underwent intensive induction therapy, had complete clinical records, and were randomly sampled. As shown in **Supplementary Table 8**, this cohort exhibited significant baseline differences from the training cohort (n = 298), including younger age (median: 49 vs. 54.5 years, *P* < 0.001; 86.3% < 60 years vs. 63.4%), higher hemoglobin levels (85 vs. 75 g/L, *P* = 0.023), distinct FAB subtype distribution (*P* < 0.001; e.g., M2: 28.8% vs. 39.3%; M5: 26.3% vs. 22.1%), different treatment patterns (65% received HSCT vs. 22.1%, *P* < 0.001; 3.8% received venetoclax vs. 19.1%, *P* < 0.001), and discordant cytogenetic risk profiles (n-2023 CN: *P* = 0.008; e.g., favorable-risk: 48.8% vs. 30.2%; intermediate-risk: 26.3% vs. 31.2%). These discrepancies reflect the inherent heterogeneity in patient demographics and institutional treatment protocols.

**Figure 5:**
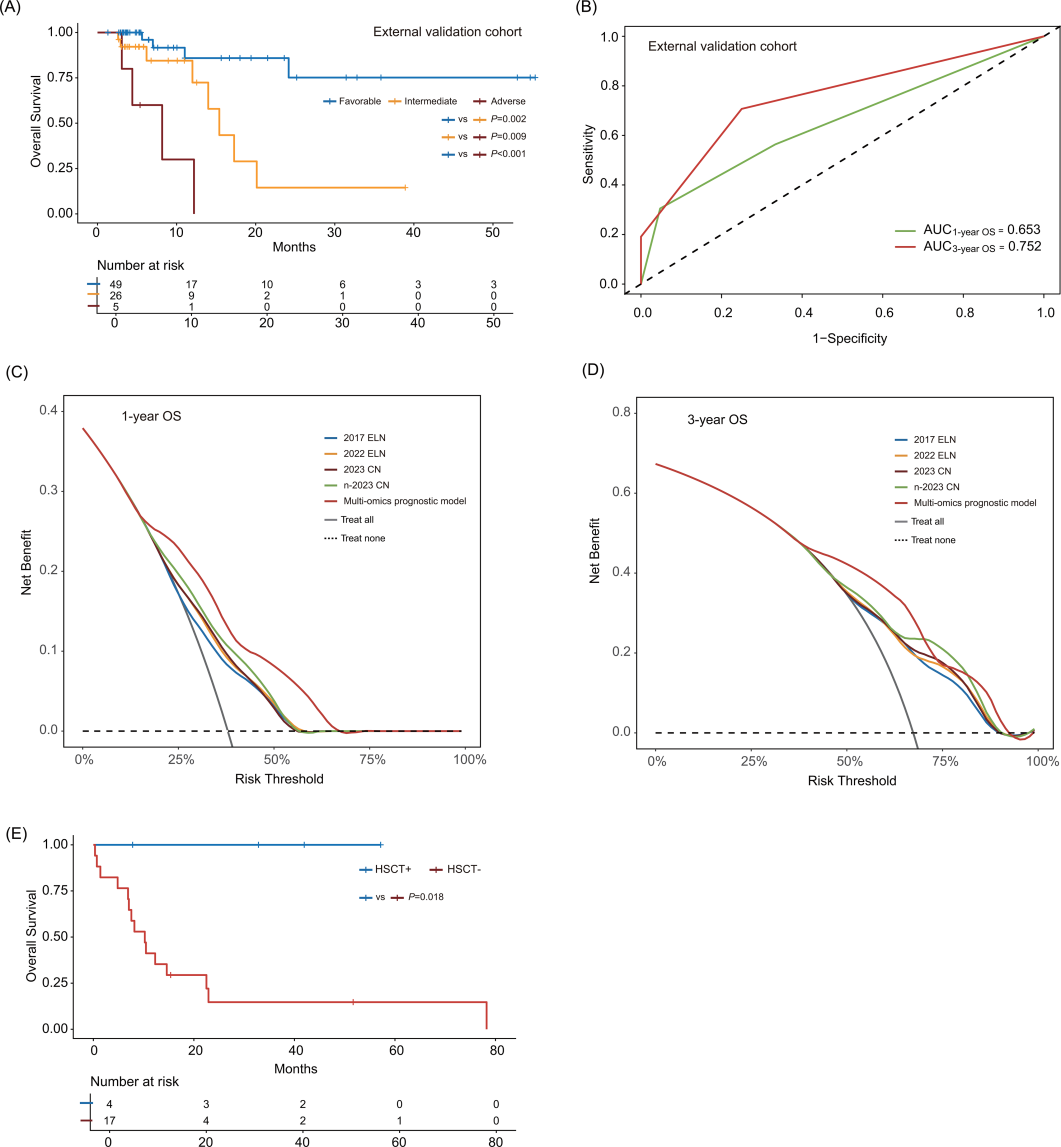
External validation and clinical translation of the multi−omics prognostic model. **(A)** Overall survival of patients in the external validation cohort according to the multi−omics prognostic model. **(B)** ROC curves of the multi−omics prognostic model for predicting 1−year and 3−year overall survival in the external validation cohort. Decision curve analysis of the multi−omics prognostic model for **(C)** 1−year OS and **(D)** 3−year OS. **(E)** Survival comparison of patients reclassified as adverse risk by n−2023 CN or the multi−omics prognostic model with and without HSCT. Fav, Favorable; Int, Intermediate; Adv, Adverse; AUC, area under the curve; HSCT, hematopoietic stem cell transplantation; OS, overall survival.

Notwithstanding these differences, the model stratified patients into favorable-risk (n = 49), intermediate-risk (n = 26), and adverse-risk (n = 5) groups. Significant survival disparities were observed among the three groups (*P* < 0.05; **Figure 5A**). The AUC values for 1-year and 3-year OS prediction were 0.653 and 0.752, respectively (**Figure 5B**). Subgroup analyses showed survival outcomes across different patient demographics and institutional practices.

To further evaluate its clinical applicability, we employed DCA to evaluate the clinical utility of four genetic risk stratifications and the multi-omics prognostic model by quantifying net benefit. Compared with the 2017 ELN, 2022 ELN, and 2023 CN, both the n-2023 CN and the multi-omics prognostic model exhibited significantly higher net benefit across a wide range of clinically relevant threshold probabilities (**Figures 5C, D**). These results confirm their dual superiority: enhanced accuracy in risk stratification and improved capacity to inform therapeutic decision-making. The findings are consistent with existing evidence, underscoring the clinical value of the novel genetic risk stratification and integrated prognostic model, which provides clinicians with a robust tool for identifying patients at high risk of adverse outcomes.

Beyond risk stratification and predictive performance, we further explored its potential to guide clinical treatment decisions. To assess whether modifications to the n-2023 CN and multi-omics prognostic model, compared with the three prior stratifications, confer tangible clinical benefits for treatment guidance, we incorporated actual clinical outcomes and survival durations into survival analyses (deviating from standard HSCT-censoring protocols).

Notably, in our study cohort, 21 patients categorized as favorable- or intermediate-risk by the 2017 ELN, 2022 ELN, and 2023 CN were reclassified as adverse-risk under the n-2023 CN or multi-omics prognostic model, with 4 undergoing HSCT. Notably, the 4 HSCT recipients had significantly longer OS than non-recipients (*P* = 0.018): all survived to the 3-year OS endpoint, whereas the 3-year OS rate among non-HSCT patients was only 14.7% (**Figure 5E**). This suggests that earlier adverse-risk identification and prompt HSCT could improve survival outcomes, validating that the n-2023 CN and multi-omics prognostic model provide meaningful clinical utility for personalized treatment guidance.

### 3.6 Comparative analysis of the multi-omics model with a published model

**Figure 6:**
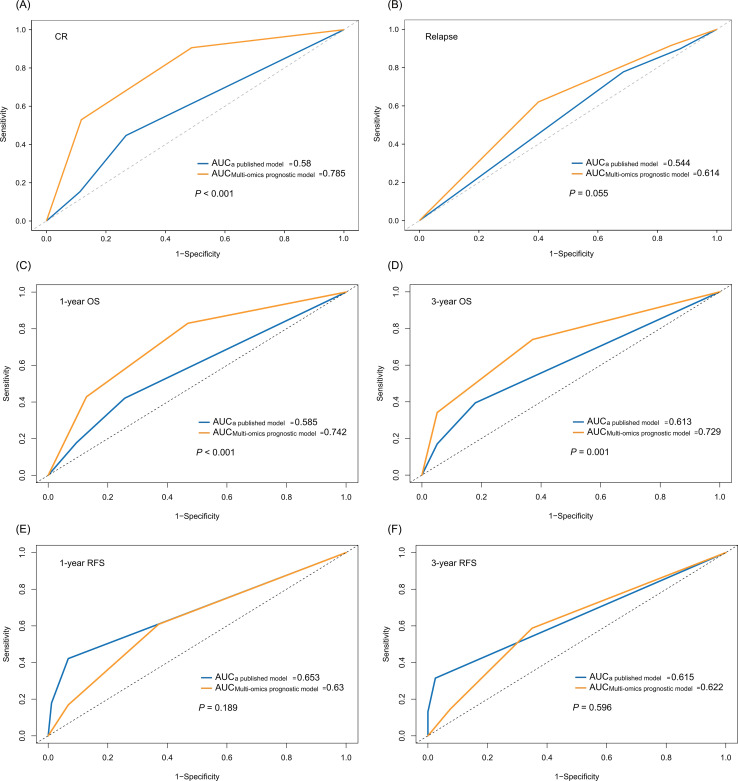
ROC curves comparing the multi-omics prognostic model with a published model. Predictive performance for **(A)** complete remission (CR), **(B)** relapse, **(C)** 1-year overall survival (OS), **(D)** 3-year OS, **(E)** 1-year relapse-free survival (RFS), and **(F)** 3-year RFS.

To compare the performance and clinical utility of our multi-omics prognostic model with an existing model, we evaluated a recently published AML prognostic model in our cohort. This model incorporates relapse-free interval after complete remission, age, white blood cell count, mutated *TP53*, *FLT3-ITD*, core-binding factor abnormalities, t(v;11q23)/*KMT2A* rearrangements, and complex/monosomal karyotype (29). Using patients’ actual survival outcomes and follow-up durations, we evaluated its predictive capacity for CR, relapse, 1-year OS, 3-year OS, and RFS, then compared these metrics with those of our multi-omics prognostic model. As depicted in **Figure 6**, our multi-omics prognostic model demonstrated superior prognostic accuracy. Significant differences were observed in the prediction of CR, 1-year OS, and 3-year OS: the AUC for CR was 0.58 for the published model versus 0.785 for ours (*P* < 0.001); for 1-year OS, AUCs were 0.585 versus 0.742 (*P* < 0.001); and for 3-year OS, 0.613 versus 0.729 (*P* =  0.001).

The published model integrates only molecular, genomic, and clinical parameters, ignoring immunophenomic and cytogenetic markers that are critical for characterizing the AML immune landscape. In contrast, our multi-omics model captures the immune-genetic interaction in AML, leading to significantly higher predictive accuracy for CR and OS. This highlights the value of multi-omics integration in exploring immune cell profiles and tumor prognosis.

## 4 Discussion

Precise risk stratification is crucial yet challenging due to AML’s heterogeneity (1). Recent studies on 18-65-year old *de novo* AML patients receiving intensive chemotherapy found that the 2017 and 2022 ELN could distinguish adjacent risk strata, consistent with our results (10). Our cohort further showed that the 2023 CN outperformed the 2017 and 2022 ELN in predicting relapse and RFS. However, no consensus exists on which systems to select for Chinese patients with specific genetic variants, given the varied outcomes.

To fill this gap, we analyzed data from Chinese AML patients receiving intensive induction therapy at two hospitals, censoring HSCT patients from transplantation dates to minimize treatment bias. Using real-world data from 298 patients, we developed the n-2023 CN stratification system. Its core innovation lies in reconstructing risk weights for genetic variants by mining clinical data tailored to the characteristics of the Chinese population, thereby resolving localization controversies in traditional systems for variants such as *CEBPA* and *FLT3-ITD*. Specifically, it adjusts risk weights for controversial variants. We propose modifications to the 2022 ELN and 2023 CN compared to the 2017 ELN: include *CEBPA*^bZIP^ (not just *CEBPA*^biallelic)^ in favorable risk, and categorize additional MR mutations (e.g., *BCOR*, *EZH2*) as adverse risk. The *CEBPA* bZIP domain is critical for both transcription factor function and regulation of hematopoiesis, and its in-frame mutations (*CEBPA*^bZIP^) are associated with favorable OS and RFS in AML patients (regardless of biallelic status). Beyond regulating hematopoiesis, this domain also mediates the transcription of immune-related genes (e.g., cytokines, chemokines)—a function supported by prior studies—that contributes to a more favorable anti-tumor biological profile compared with *CEBPA*^biallelic (only)^ patients. This dual mechanism underscores the value of prioritizing *CEBPA*^bZIP^ in risk stratification, as it enables precise identification of low-risk patients and reduces the potential for unnecessary overtreatment (22, 23). MR genes involve chromatin remodeling, splicing, and DNA repair (30); mutations disrupt these processes, driving AML progression (31), and impairing the function of anti-tumor immune cells including T cells and NK cells, which supports the classification of MR gene-mutated patients as adverse-risk (32, 33). This optimization aids early initiation of strategies like allogeneic HSCT in adverse-risk patients.

Additionally, *FLT3* mutation activate aberrant signaling, and high-load *FLT3-ITD* (*FLT3-ITD*^high^) correlates with chemotherapy resistance, high post-CR relapse rates, and shorter OS; it can also induce an immunosuppressive microenvironment by promoting the secretion of cytokines such as IL-10 and TGF-β, impairing NK-cell cytotoxicity, and facilitating immune escape (34–37). However, its risk allocation has been revisited in the 2022 ELN update, which classifies all *FLT3-ITD* mutated cases as intermediate risk regardless of allelic ratio or *NPM1* co-mutation, largely reflecting the expected survival benefits of *FLT3* inhibitors in healthcare systems where these agents are widely available and routinely incorporated into frontline regimens (38, 39). In contrast, both our data and recent real-world studies from China and other Asian countries indicate that this premise is not yet fulfilled in our setting. A national, registry-based, real-world study from China showed that *FLT3* inhibitors are used in only a minority of patients due to delayed approval, restricted reimbursement, and substantial out-of-pocket costs (40). Consistently, phase 3 and long-term follow-up analyses of predominantly Asian patients with relapsed/refractory *FLT3*-mutated AML in the COMMODORE program have emphasized that limited access to newly approved targeted agents and delayed implementation in routine care remain major challenges across Asian healthcare systems, potentially leaving many eligible patients undertreated (41, 42). Notably, gilteritinib only received conditional approval in China in February 2021, further illustrating the temporal gap between guideline recommendations and real-world availability (10, 43). Our cohort reflects this situation. Only 30.2% (13 of 43) of evaluable *FLT3-ITD* mutated patients actually received *FLT3* inhibitors, although all received intensive chemotherapy. In the absence of inhibitors, *FLT3-ITD*, whether with a high or low allelic ratio and with or without *NPM1* co-mutation, was associated with significantly worse OS than the intermediate-without-variants group. In contrast, among patients who received inhibitors, OS was comparable to that of the group (**Supplementary Figure 4**). These findings indicate that the adverse impact of *FLT3-ITD* on OS is largely unmitigated when targeted therapy is not used, which remains the real-world scenario for most Chinese patients. Moreover, recent mechanistic and clinical studies show that *FLT3-ITD*, particularly at high allelic burden, is linked to clonal dominance, genomic instability, and a high propensity to develop resistance via secondary *FLT3-TKD* mutation or activation of alternative pathways, and thus continues to confer a poor prognosis even in the targeted era (44). Taken together, these data support retaining the *FLT3-ITD* allelic ratio in the n-2023 CN stratification and classifying *FLT3-ITD*^high^ as adverse risk and *FLT3-ITD*^low^ as intermediate risk specifically in the absence of *FLT3* inhibitors. This design allows the n-2023 CN to function as a conditionally adaptable framework. In settings where *FLT3* inhibitors are not universally available or affordable, the allelic ratio remains essential to avoid underestimating the risk of *FLT3-ITD* mutated patients and to prioritize them for early allogeneic HSCT. At the same time, the model can be recalibrated as targeted therapies become more widely implemented.

*KIT* mutation occurs in 4-6% of adult *de novo* AML and 20-40% of CBF-AML, most commonly at the D816 locus (45). Chinese CBF-AML patients have a higher *KIT*^D816^ mutation rate than those in Japan, South Korea, the US, and other Western countries (46–49). While the 2022 ELN classifies *KIT*-mutated CBF-AML as favorable-risk, *KIT*^D816^ activates SRC and JAK-STAT pathways, enhancing proliferation (50–52). Our study confirms that CBF-AML with *KIT*^D816^ has comparable OS but worse RFS vs. variant-free intermediate-risk cases, supporting 2023 CN’s localized reclassification to intermediate-risk—a precision medicine distinction from Western systems. The n-2023 CN outperforms existing stratifications in predictive efficacy across metrics.

Beyond genetics, immunophenotypic markers and clinical parameters affect prognosis. We integrated 49 variables to identify independent OS risk factors. CD15 (Lewis X), a fucosylated antigen, is expressed on diverse cancer cells (53–59). In prostate cancer, its O-glycan reduces NK cell-cancer cell interaction, weakening cytotoxicity and enabling immune evasion (53, 60). In thyroid cancer, CD15 has been recognized as a cancer stem cell marker (61). its fucose moiety binds selectins, promoting adhesion and metastasis (62). In AML, CD15’s prognostic role is inconsistent, with studies that include or exclude APL reporting varying associations (13, 14, 63). Several studies have indicated that CD15 is linked to adverse clinical outcomes in AML: it correlates with higher frequencies of *DNMT3A* and *FLT3-ITD* mutations, as well as elevated Bcl-2 protein levels, and is associated with inferior responses to standard induction chemotherapy and shorter OS (12–15). Our study found CD15 positivity independently predicts poorer OS, linked to higher initial bone marrow blasts, peripheral leukocytes, and post-CR relapse rates (**Supplementary Table 7**). Our *in vitro* functional assays further validated the pro-oncogenic effects of CD15, demonstrating that CD15 directly enhances the proliferative, migratory, and invasive capacities of AML cells. Moreover, blocking CD15 epitopes with specific antibodies effectively inhibited the proliferative activity and invasive/migratory potential of CD15-positive AML cells (64). Concurrently, our study revealed that CD15 positivity significantly reduces the proportion of NK cells in the peripheral immune microenvironment and impairs the body’s innate anti-leukemic immune response. This immune evasion mechanism is consistent with the CD15-mediated regulatory mechanism identified in prostate cancer, and this effect may compromise the immune system’s ability to eliminate minimal residual leukemic cells, thereby increasing the risk of relapse in patients after CR. Furthermore, published studies have confirmed that the fucosyltransferase 4 (FUT4) gene, which modulates CD15 biosynthesis, can upregulate the malignant behaviors of leukemic stem cells (LSCs) in AML via the MiR-29b/Sp1/FUT4 signaling axis. Aberrant maintenance of stemness in LSCs represents the core molecular basis for differentiation arrest in AML cells (65). This suggests that CD15 can regulate the stemness maintenance, self-renewal, and malignant proliferation of LSCs, which not only contributes to the development of differentiation arrest in AML cells but also mediates leukemogenesis, disease progression, and relapse. These findings provide critical mechanistic insights into the markedly elevated relapse rate observed in CD15-positive patients in our study and further establish a close association between CD15 and LSC biological characteristics, as well as cellular differentiation arrest in AML. Collectively, CD15 drives AML disease progression and ultimately leads to poor clinical outcomes in patients through multiple mechanisms, including immune suppression, enhancement of tumor cell invasiveness, modulation of LSC stemness, and mediation of cellular differentiation arrest.

Additionally, chromosomal abnormalities like monosomy seven (-7) and del(7q), which occur in up to 10% of AML patients, affect progression and treatment sensitivity (31). They may synergize with CD15 positivity to worsen risk.

Age profoundly impacts AML prognosis (9, 66), with Mrozek et al. advocating for age-stratified ELN genetic risk classification (6). In our n-2023 CN adverse-risk subgroup, age predicts outcomes: 3-year OS was 4.7% for ≥ 60 vs. 28.3% for < 60, highlighting its utility in treatment stratification. These three factors synergize to define adverse phenotypes.

The multi-omics prognostic model (integrating age, CD15 positivity, and del(7q)) demonstrated robust performance in an external validation cohort at a tertiary hospital 300 km away, thereby ensuring spatial and institutional diversity. Despite differences in age, baseline characteristics, and treatment between cohorts, the consistency indicates reliance on universal biological signatures rather than institutional or population biases, supporting real-world applicability across settings. Subsequently, we incorporated the actual survival status and survival time of patients who received HSCT into the analysis. Notably, among patients reclassified as adverse-risk by the n-2023 CN or multi-omics prognostic model despite being initially stratified as favorable- or intermediate-risk by traditional systems, those who received HSCT demonstrated significantly improved survival compared with their non-transplant counterparts. This finding confirms the prognostic accuracy of n-2023 CN and the new model, underscoring their value in optimizing treatment decisions. In terms of clinical feasibility, the new model does not impose additional economic burden compared to the original genetic stratification. For example, del(7q) is included in chromosomal karyotype analysis for genetic stratification, CD15 is a mandatory item in disease typing diagnosis, and age acquisition incurs no extra cost or technical threshold.

Our model has superior predictive accuracy, with higher AUCs than a published AML model for CR, 1-year OS, and 3-year OS, highlighting its value for Chinese patients. The published model includes classic genetic markers (e.g., *FLT3-ITD*, *TP53*) and clinical parameters (e.g., age, white blood cell count), but performs poorly in our cohort, especially for CR. This discrepancy likely stems from the underrepresentation of region-specific genetic or immunophenotypic features in the published model. Our inclusion of del(7q) and CD15, confirmed here as independent adverse factors, fills this gap: del(7q) is more prevalent in Asian AML and is linked to chemotherapy resistance; CD15, as an immune evasion marker, correlates with higher relapse rates. These variables, absent from the published model, capture the biological heterogeneity of Chinese AML.

Our study has several strengths, including a multicenter cohort covering a wide age range and the administration of uniform intensive induction chemotherapy. However, several limitations should be acknowledged. First, the retrospective design and relatively modest sample size may reduce statistical power in analyzing rare genetic variants, limiting the ability to draw definitive conclusions about their independent prognostic impact. Second, although our external validation cohort was established using stratified random sampling to reduce selection bias, its single-center origin inevitably introduces geographic and institutional heterogeneity; notably, patients in this cohort were younger, harbored more favorable-risk genetics, and had a substantially higher transplantation rate (65% vs. 22.1%). To minimize confounding, all patients undergoing HSCT were censored at the time of transplantation in validation analyses. Third, only 5 patients in the external cohort were stratified into the adverse-risk group by our multi-omics model, which restricts the robustness of subgroup analyses in this category. Fourth, patient enrollment spanned 2015–2023 and was initially based on the 2016 WHO classification; although a retrospective review confirmed that all cases met the 2022 WHO/ICC AML criteria, some early-stage patients with low blast counts (< 20%) and *NPM1* or *CEBPA* mutations were not included (67, 68). In addition, the requirement for intensive induction chemotherapy inherently selects for medically fit patients, and complete-case analysis may exclude those with rapid disease progression, representing potential selection bias. Finally, our model is specifically validated for fit patients receiving intensive chemotherapy and cannot be directly extrapolated to unfit populations treated with low-intensity regimens such as hypomethylating agents combined with venetoclax. Future large-scale, prospective, multicenter studies are therefore warranted to comprehensively validate our findings across diverse AML populations.

In conclusion, we introduced a new genetic stratification and prognostic model integrating genetic, immunological, and clinical parameters. These findings advance research and practice. With a deeper understanding of AML’s molecular pathology, we anticipate more effective treatments and improved survival.

## Data Availability

The raw data supporting the conclusions of this article will be made available by the authors, without undue reservation.
