# Supplementary material for: Multi-omics integration of molecular genetics, cytogenetics and immunophenotyping: a novel prognostic model for immune landscape characterization and outcome prediction in Chinese patients with acute myeloid leukemia
Source: Front Immunol. 2026 May 1;17:1812970. doi: 10.3389/fimmu.2026.1812970 (PMC13175975; doi:10.3389/fimmu.2026.1812970)
Supplement: Supplementary file 1 [file Supplementaryfile1.doc]

# *Supplementary Materials*

# 1 Supplementary Methods

1.1 Patients and treatment

All patients were diagnosed with acute myeloid leukemia per the World Health Organization’s 2016 hematolymphoid neoplasm criteria,^[69]^ aged ≥ 15 years at initial diagnosis, excluding those with acute promyelocytic leukemia (APL). All received intensive induction chemotherapy via a '3 + 7' IA/DA-based regimen (IA: Idarubicin + Cytarabine; DA: Daunorubicin + Cytarabine): idarubicin or daunorubicin for the first 3 days, and cytarabine for the initial 7 days. This 'based regimen' encompasses both standard-dose protocols for younger/fit patients and appropriately dose-attenuated or age-adjusted intensive protocols for elderly patients (such as reduced doses of anthracyclines and cytarabine). Importantly, patients who received non-intensive alternatives to frontline induction (e.g., standalone hypomethylating agents and/or targeted therapies without an intensive chemotherapy backbone) were excluded to maintain treatment homogeneity. For the subset of patients who received venetoclax (n = 67), the drug was administered strictly as an adjunct to chemotherapy, only after induction therapy, including consolidation therapy, maintenance therapy, or relapse/salvage therapy. Additionally, due to the study's broad temporal span (2015-2023) and limited availability of targeted *FLT3* inhibitors in China in earlier years, these agents were administered to only a subset of eligible patients during their treatment courses. Post-complete remission (CR), patients underwent consolidation chemotherapy. Collected data included sex, age at diagnosis, hematological and biochemical results, bone marrow (BM) blast percentage, cytomorphological classification, immunophenotypic analysis, chromosomal karyotype, next-generation sequencing (NGS) mutation profiles, remission status, relapse information, and survival time. Telephone follow-up supplemented data for patients treated elsewhere. Regarding the management of missing data, a complete-case analysis approach was strictly employed. As defined in our inclusion and exclusion criteria, individuals lacking comprehensive baseline clinical, flow cytometric, cytogenetic, or molecular data were excluded prior to analysis. This rigorous patient selection ensured a fully populated dataset for all required variables in the final modeling and validation cohorts.

A total of 378 eligible patients were enrolled between April 2015 and May 2023 from three hospitals. The study included a training cohort and an external validation cohort. The training cohort consisted of 298 patients (98 from the Second Hospital of Anhui Medical University, 200 from the First Affiliated Hospital of the University of Science and Technology of China), which was used to assess the prognostic efficacy of the 2017 ELN, 2022 ELN, and 2023 CN stratifications, re-evaluate controversial genetic variants, and establish the novel n-2023 CN stratification. Multivariate Cox regression was performed to identify independent prognostic factors and construct a multi-omics prognostic model with internal validation.

The external validation cohort comprised 80 patients from the First Affiliated Hospital of Soochow University. To mitigate potential selection and temporal biases, we employed a chronologically stratified random sampling approach rather than convenience sampling. Specifically, eligible patients were stratified chronologically by their admission period using medical record serial numbers, and random sampling was performed proportionally within each temporal stratum to capture the natural clinical heterogeneity of the hospital’s AML population across the study timeframe. Crucially, the eligibility criteria for this external validation cohort were designed to be strictly identical to those of the training cohort. The inclusion criteria were: (1) newly diagnosed AML (excluding APL) per the 2016 WHO criteria; (2) aged 15 years or older at initial diagnosis; (3) received intensive induction chemotherapy via a '3+7' IA/DA-based regimen; and (4) possessed complete baseline clinical, cytogenetic, molecular, and immunophenotypic data. The exclusion criteria included patients receiving non-intensive induction therapies (such as standalone hypomethylating agents and/or targeted therapies without an intensive chemotherapy backbone), targeted monotherapies, or those with missing key baseline data.

This study was approved by the Institutional Review Board of the participating centers (Approval No.: YX2024-120), and all procedures were performed in accordance with the Declaration of Helsinki.

Given the significant prognostic impact of hematopoietic stem cell transplantation (HSCT), patients were censored at HSCT during risk stratification validation, construction, or validation of the n-2023 CN, and model development. However, when assessing whether the new stratification or model could guide beneficial treatment, or when comparing it with a published model, the actual survival outcomes and times of HSCT recipients were reincluded.

1.2 Multi-omics profiling: molecular genomics, cytogenomics, and immunophenomics testing

Genetic and immunologic assessments used BM samples from the initial diagnosis. NGS detected 30 common AML-associated mutations (*ASXL1, BCOR, BCORL1, CALR, CBL, CEBPA, CSF3R, DNMT3A, ETV6, EZH2, FLT3, IDH1, IDH2, JAK2, KIT, KRAS, MPL, NPM1, NRAS, PHF6, PIGA, RUNX1, SETBP1, SF3B1, SRSF2, TET2, TP53, U2AF1, WT1*, and *ZRSR2*). Chromosomal karyotyping analyzed 20 metaphase cells (24-hour culture) via G-banding. Immunophenotyping was performed using 8-10 color flow cytometry (50,000–100,000 cells) to detect a standardized panel of leukemia-associated antigens. Antigens were considered positive if expressed on ≥ 10% of gated blasts (regardless of surface or intracellular location).^[70]^

1.3 Definitions and statistical analysis

CR and relapse were defined per 2017 ELN guidelines.^[2]^ Overall survival (OS) was measured from the time of diagnosis to death, end of follow-up, or HSCT. Relapse-free survival (RFS) was calculated from the time of initial CR to first relapse, death, end of follow-up, or HSCT.

Categorical variables were compared using the chi-square or Fisher’s exact tests, and continuous variables were compared between groups via the Kruskal-Wallis test. Kaplan-Meier analysis (Log-rank test) was used to evaluate differences in survival. Variables significant at *P* < 0.05 in univariate Cox proportional hazards regression analysis were selected as candidate variables. To rule out potential multicollinearity among these candidate variables, variance inflation factor (VIF) analysis was performed, with a VIF < 5 indicating the absence of significant multicollinearity. Subsequently, a multivariate Cox proportional hazards regression model was constructed to identify independent prognostic factors. Model stability was validated via LASSO-Cox regression. Predictive accuracy was assessed via the area under the receiver operating characteristic curve (AUC), with pairwise comparisons using DeLong’s test. Internal validation was performed using bootstrap resampling (1,000 iterations); calibration curves were used to evaluate predicted vs. observed probabilities of 1-, 2-, and 3-year OS, with C-index as the metric. Decision curve analysis (DCA) was used to assess clinical utility. Optimal cut-offs for continuous variables and cumulative multi-omics risk scores were determined using X-tile software. Analyses and graphics were performed using R software (version 4.2.0).

# 2 Supplementary Figures

**Figure S1** ROC curves of the 2017 ELN, 2022 ELN, and 2023 CN.

**(A)** ROC curve of the 2017 ELN, 2022 ELN, and 2023 CN in predicting CR, **(B)** relapse, **(C)** 1-year OS, **(D)** 3-year OS, **(E)** 1-year RFS, **(F)** 3-year RFS for patients.

**Figure S2** Overall survival of patients with specific genetic variants in the 2017 ELN, 2022 ELN and 2023 CN.

**Abbreviations**: t(8;21)/inv(16)-*KIT*^D816^, t(8;21)(q22;q22) or inv(16)(p13q22)/t(16;16)(p13;q22) with mutated *KIT*^D816^; t(8;21)/inv(16), t(8;21)(q22;q22) or inv(16)(p13q22)/t(16;16)(p13;q22) without mutated *KIT*^D816^; *FLT3-ITD(±NPM1)*, mutated *FLT3-ITD* (regardless of whether it is combined with *NPM1* mutation); *FLT3-ITD-NPM1*, mutated *FLT3-ITD* and *NPM1*.

**Figure S3** Relapse-free survival of patients with specific genetic variants in the 2017 ELN, 2022 ELN and 2023 CN.

**Figure S4** Overall and relapse-free survival of patients with mutated *FLT3-ITD* and receiving inhibitors treatment.

**Abbreviations**: inhi+, receiving *FLT3* inhibitors treatment; inhi-, not receiving *FLT3* inhibitors treatment.

**Figure S5** ROC curves of the 2017 ELN, 2022 ELN, 2023 CN, n-2023 CN and multi-omics prognostic model.

**(A)** ROC curve of the 2017 ELN, 2022 ELN, 2023 CN, n-2023 CN and multi-omics prognostic model in predicting CR, **(B)** relapse, **(C)** 1-year OS, **(D)** 3-year OS, **(E)** 1-year RFS, **(F)** 3-year RFS for patients. AUC, areas under the curve.

**Figure S6** Correlation between CD15 expression levels and peripheral blood lymphocyte subset distribution in AML patients.

**Abbreviations**: TREG, regulatory T cells; TEF, effector T cells; NK cells, natural killer cells; #, absolute count.

# 3 Supplementary Tables

**Table S1** Patients with different genetic risks according to the 2017 ELN and 2022 ELN.

| **2017 ELN** | **2022 ELN** | **N** |
| --- | --- | --- |
| **Favorable** | **Intermediate** | **25** |
| Mutated *NPM1* with *FLT3-ITD*^low^ | Mutated *NPM1* and *FLT3-ITD* | 19 |
| Mutated *NPM1* with *FLT3-ITD*^low^ | Mutated *NPM1*, *FLT3-ITD*, and *BCOR* | 3 |
| Mutated *NPM1* with *FLT3-ITD*^low^ | Mutated *NPM1*, *FLT3-ITD*, and *U2AF1* | 1 |
| Biallelic mutated *CEBPA*, Mutated *FLT3-ITD*^low^ | Mutated *FLT3-ITD* | 1 |
| Biallelic mutated *CEBPA* | Genetic variants not classified as favorable or adverse | 1 |
|  | **Adverse** | **1** |
| Biallelic mutated *CEBPA* | Mutated *BCOR* | 1 |
| **Intermediate** | **Favorable** | **2** |
| Mutated *FLT3-ITD*^low^ | bZIP in-frame mutated *CEBPA* and mutated *FLT3-ITD* | 2 |
|  | **Adverse** | **14** |
| Mutated *FLT3-ITD*^low^ | Mutated *FLT3-ITD*, *BCOR*, and *SRSF2* | 1 |
| Genetic variants not classified as favorable or adverse | Mutated *BCOR* | 6 |
| Genetic variants not classified as favorable or adverse | Mutated *SF3B1* | 2 |
| Genetic variants not classified as favorable or adverse | Mutated *SRSF2* | 2 |
| Genetic variants not classified as favorable or adverse | Mutated *U2AF1* | 1 |
| Genetic variants not classified as favorable or adverse | Mutated *ZRSR2* | 1 |
| Genetic variants not classified as favorable or adverse | Mutated *BCOR* and *U2AF1* | 1 |
| **Adverse** | **Favorable** | **3** |
| inv(16)(p13.1q22) with mutated *TP53* | inv(16)(p13.1q22) with mutated *TP53* (VAF<10%) | 1 |
| Biallelic mutated *CEBPA*, mutated *FLT3-ITD*^high^ | bZIP in-frame mutated *CEBPA*, mutated *FLT3-ITD* | 2 |
|  | **Intermediate** | **8** |
| Mutated *FLT3-ITD*^high^ | Mutated *FLT3-ITD* | 7 |
| Mutated *TP53* | Mutated *TP53* (VAF < 10%) | 1 |

**Abbreviations**: *FLT3-ITD*^low^*, FLT3-ITD* with low allele ratio (< 0.5); *FLT3-ITD*^high^, *FLT3-ITD* with high allele ratio (≥ 0.5); VAF, variant allele fraction.

**Table S2** Patients with different genetic risks according to the 2022 ELN and 2023 CN.

| **2022 ELN** | **2023 CN** | **N** |
| --- | --- | --- |
| **Favorable** | **Intermediate** | **9** |
| t(8;21)(q22;q22.1) | t(8;21)(q22;q22.1) with mutated *KIT*^D816^ | 8 |
| inv(16)(p13.1q22) | inv(16)(p13.1q22) with mutated *KIT*^D816^ | 1 |
|  | **Adverse** | **1** |
| inv(16)(p13.1q22) with mutated *TP53* (VAF < 10%) | inv(16)(p13.1q22) with mutated *TP53* | 1 |
| **Intermediate** | **Favorable** | **23** |
| Mutated *NPM1* and *FLT3-ITD* | Mutated *NPM1* with *FLT3-ITD*^low^ | 19 |
| Mutated *NPM1*, *FLT3-ITD*, and *U2AF1* | Mutated *NPM1*, *FLT3-ITD*^low^, and *U2AF1* | 1 |
| Mutated *NPM1*, *FLT3-ITD*, and *BCOR* | Mutated *NPM1*, *FLT3-ITD*^low^, and *BCOR* | 3 |
|  | **Adverse** | **10** |
| Mutated *NPM1*, *FLT3-ITD*, and *SRSF2* | Mutated *NPM1*, *FLT3-ITD*^high^, and *SRSF2* | 1 |
| Mutated *NPM1*, *FLT3-ITD*, and *U2AF1* | Mutated *NPM1*, *FLT3-ITD*^high^, and *U2AF1* | 1 |
| Mutated *FLT3-ITD* | Mutated *FLT3-ITD*^high^ | 7 |
| Mutated *TP53* (VAF < 10%) | Mutated *TP53* | 1 |

**Abbreviations**: *KIT*^D816^, the D816 locus of *KIT.*

**Table S3 Clinical outcomes of patients according to the 2017 ELN, 2022 ELN, and 2023 CN.**

|  | | **CR rate (%)** | **Relapse rate (%)** | **Median OS (months)** | **Median RFS (months)** | **OS (%, 95% CI)** | | | | **RFS (%, 95% CI)** | | | |
| --- | --- | --- | --- | --- | --- | --- | --- | --- | --- | --- | --- | --- | --- |
|  |  |  |  |  |  | **1-year** | | **3-year** | | **1-year** | | **3-year** | |
| **All patients** | | 71.5 | 49.3 | 17.8 | 15 | 62.1 | 0.564-0.683 | 32.7 | 0.269-0.397 | 58.7 | 0.516-0.667 | 37.9 | 0.308-0.467 |
| **2017 ELN** | **Fav** | 82.8 | 42.6 | 31 | 29 | 73.3 | 0.654-0.822 | 49 | 0.394-0.608 | 63 | 0.533-0.746 | 47.9 | 0.376-0.61 |
|  | **Int** | 77.5 | 53.6 | 20 | 15 | 63.1 | 0.532-0.749 | 29.7 | 0.204-0.433 | 60.3 | 0.487-0.746 | 33 | 0.221-0.492 |
|  | **Adv** | 49.4 | 58.1 | 9.37 | 11.4 | 44.5 | 0.343-0.578 | 12.5 | 0.063-0.246 | 44.3 | 0.294-0.667 | 22.1 | 0.11- 0.445 |
| **2022 ELN** | **Fav** | 86.1 | 44.8 | 31 | 29 | 76.3 | 0.68-0.855 | 49.8 | 0.396-0.627 | 61.5 | 0.512-0.739 | 46.4 | 0.357-0.603 |
|  | **Int** | 77.4 | 50 | 20.8 | 15.2 | 62.1 | 0.528-0.731 | 34 | 0.247-0.466 | 61.9 | 0.51-0.75 | 38.1 | 0.273-0.531 |
|  | **Adv** | 48.4 | 56.8 | 9.2 | 11.8 | 44.2 | 0.341-0.573 | 11.5 | 0.058-0.228 | 46.2 | 0.311-0.686 | 19.3 | 0.089-0.419 |
| **2023 CN** | **Fav** | 82.5 | 39.4 | 31 | 39.3 | 76.5 | 0.687-0.853 | 49.6 | 0.398-0.619 | 64.7 | 0.545-0.767 | 51.2 | 0.404-0.649 |
|  | **Int** | 82.9 | 52.9 | 20.8 | 14.2 | 61.3 | 0.509-0.738 | 33.6 | 0.235-0.48 | 61.7 | 0.502-0.759 | 34.4 | 0.234-0.508 |
|  | **Adv** | 50 | 62.7 | 9.37 | 10 | 45.6 | 0.361-0.577 | 12.2 | 0.065-0.231 | 42.7 | 0.292-0.625 | 18.3 | 0.09-0.374 |

**Abbreviations**: Fav, Favorable; Int, Intermediate; Adv, Adverse.

**Table S4 Outcomes of patients with specific genetic variants in the 2017ELN, 2022 ELN and 2023 CN.**

|  | **CR rate, % (n/N)** | ***P* value^†^** | **Relapse rate, % (n/N)** | ***P* value^†^** |
| --- | --- | --- | --- | --- |
| **The rest Fav (without *TP53*, *FLT-ITD*^high^, *KIT*^D816^)** | 85.5% (59/69) |  | 42.4% (25/69) |  |
| *CEBPA*^bZIP(/+biallelic)^ | 90% (18/20) | 1 | 38.9% (7/18) | 1 |
| *CEBPA*^biallelic(/+bZIP)^ | 95.5% (21/22) | 0.285 | 47.6% (10/21) | 0.799 |
| *CEBPA*^bZIP(only)^ | 0 | **0.027** | / | / |
| *CEBPA*^biallelic(only)^ | 75% (3/4) | 0.487 | 100% (3/3) | 0.087 |
| **Intermediate (without any variants)** | 90.4% (47/52) |  | 46.8% (22/47) |  |
| t(8;21)/inv(16)-*KIT*^D816^ | 77.8% (7/9) | 0.273 | 71.4% (5/7) | 0.42 |
| t(8;21)/inv(16) | 97.2% (35/36) | 0.394 | 45.7% (16/35) | 1 |
| **Intermediate (without any variants)** | 90.4% (47/52) |  | 46.8% (22/47) |  |
| *(NPM1-)FLT3-ITD* | 62.8% (27/43) | **0.002** | 51.9% (14/27) | 0.81 |
| *NPM1-FLT3-ITD* | 62.5% (20/32) | **0.004** | 40% (8/20) | 0.789 |
| *(NPM1-)FLT3-ITD*^high^ | 65% (13/20) | **0.016** | 69.2% (9/13) | 0.213 |
| *(NPM1-)FLT3-ITD*^low^ | 60.9% (14/23) | **0.008** | 35.7% (5/14) | 0.549 |
| *NPM1-FLT3-ITD*^high^ | 69.2% (9/13) | 0.07 | 55.6% (5/9) | 0.725 |
| *NPM1-FLT3-ITD*^low^ | 57.9% (11/19) | **0.004** | 27.3% (3/11) | 0.32 |
| **MR genes without any other variants in Fav2022** | 45.5% (25/55) |  | 60% (15/25) |  |
| MR genes with some other variants in Fav2022 | 80% (12/15) | **0.021** | 25% (3/12) | 0.079 |
| **Intermediate (without any variants)** | 90.4% (47/52) |  | 46.8% (22/47) |  |
| MR genes (without any other variants) | 53.8% (7/13) | **0.006** | 71.4% (5/7) | 0.42 |
| ***TP53* (VAF<10%)** | 100% (2/2) |  | 100% (2/2) |  |
| *TP53* (VAF ≥10%) | 35.3% (6/17) | 0.164 | 50% (3/6) | 0.464 |
| Intermediate (without any variants) | 90.4% (47/52) | 1 | 46.8% (22/47) | 0.235 |

**Abbreviations**: **^†^**, the *P* value obtained by comparing with the previous subheading (in bold); Fav, Favorable; t(8;21)/inv(16), t(8;21)(q22;q22)/ inv(16)(p13q22)/t(16;16)(p13;q22); t(8;21)/inv(16)-*KIT*^D816^, t(8;21)(q22;q22)/ inv(16)(p13q22)/t(16;16)(p13;q22) with mutated *KIT*^D816^; *NPM1-FLT3-ITD*, mutated *NPM1* and *FLT3-ITD*; Fav2022, favorable-risk group in the 2022 ELN.

**Table S5 Outcomes of patients according to the n-2023 CN and multi-omics prognostic model.**

|  |  | **N (%)** | **CR rate (%)** | **Relapse rate (%)** | **Median OS (months)** | **Median RFS (months)** |
| --- | --- | --- | --- | --- | --- | --- |
| **All Patients** |  |  | 71.5 | 49.3 | 17.8 | 15 |
| **The n-2023 CN** | **Favorable** | 90 (30.2) | 86.7 | 42.3 | 48.1 | 29.4 |
|  | **Intermediate** | 93 (31.2) | 80.6 | 48 | 22.4 | 17.7 |
|  | **Adverse** | 115 (38.6) | 52.2 | 60 | 10.2 | 9.73 |
| **The** **multi-omics prognostic model** | **Favorable** | 117 (39.3) | 93.2 | 38.5 | Not reached | 39.3 |
|  | **Intermediate** | 111 (37.2) | 71.2 | 59.5 | 13.0 | 11.4 |
|  | **Adverse** | 70 (23.5) | 35.7 | 64 | 5.87 | 8.8 |

**Abbreviations**: CR, complete remission; OS, overall survival; RFS, Relapse-free survival.

**Table S6** Variance inflation factors assessing multicollinearity in multivariable Cox model.

| **Variable** | **VIF value** | **Df** | **Interpretation** |
| --- | --- | --- | --- |
| **Age, years** | 1.12 | 1 | No collinearity |
| **WBC, 10^9^/L** | 1.21 | 1 | No collinearity |
| **Origin of disease** | 1.26 | 1 | No collinearity |
| **Gene mutations count** | 1.36 | 1 | No collinearity |
| ***DNMT3A*** | 1.17 | 1 | No collinearity |
| ***IDH2*** | 1.14 | 1 | No collinearity |
| ***TET2*** | 1.20 | 1 | No collinearity |
| **CD15** | 1.09 | 1 | No collinearity |
| **CD19** | 1.14 | 1 | No collinearity |
| **del(7q)** | 1.10 | 1 | No collinearity |
| **n-2023 CN** | 1.15 | 2 | No collinearity |

**Abbreviations**: VIF, variance inflation factor; Df, degrees of freedom. VIF < 5 indicates no substantial multicollinearity between variables. For the three-level categorical variable n-2023 CN (Df = 2), the equivalent VIF (calculated as [Generalized variance inflation factor^(1/(2*Df))]^2) = 1.15, confirming no multicollinearity.

**Table S7** Characteristics of patients with **CD15 positive and negative.**

| **Characteristics** | **CD15 positive**  **(n = 80)** | **CD15 negative**  **(n = 218)** | ***P* value** |
| --- | --- | --- | --- |
| **Age, years** | 54.5 (15-79) | 54.5 (15-81) | 0.664 |
| ≥ 60 | 30 (37.5%) | 79 (36.2%) | 0.892 |
| **Gender (male/female)** | 42/38 | 103/115 | 0.501 |
| **BM blasts, %** | 69.4 (17-96.5) | 53.25 (3.5-97) | **0.002** |
| **WBC, 10^9^/L** | 25.62 (0.4-440) | 10.64 (0.55-385.29) | **0.002** |
| **HB, g/L** | 76.5 (39-138) | 75 (32-130) | 0.727 |
| **PLT, 10^9^/L** | 40.5 (3-395) | 40 (3-485) | 0.801 |
| **FAB-classification** |  |  | **< 0.001** |
| M0 | 0 | 2 (1.2%) |  |
| M1 | 3 (4.3%) | 14 (8.1%) |  |
| M2 | 21 (30.4%) | 96 (55.8%) |  |
| M4 | 12 (17.4%) | 26 (15.1%) |  |
| M5 | 33 (47.8%) | 33 (19.2%) |  |
| M6 | 0 | 1 (0.6%) |  |
| Not available | 11 (13.8%) | 46 (21.1%) |  |
| **Origin of disease** |  |  |  |
| De novo / S-AML | 76/4 | 207/11 | 1 |
| **CR** | 55 (68.8%) | 168 (77.1%) | 0.189 |
| **Relapse** | 36 (65.5%) | 83 (49.4%) | **0.04** |
| **Extramedullary disease** | 14 (17.5%) | 15 (6.9%) | **0.018** |

**Abbreviations**: BM, bone marrow; WBC, white blood cell; HB, hemoglobin; PLT, platelet; FAB, French-American-British; S-AML, secondary AML; CR, complete remission. Note: Percentages for FAB classification (M0-M6) are calculated based on the number of patients with available FAB classification data, excluding patients with not available status.

**Table S8** Baseline characteristics of training and external cohort.

|  | **Training cohort**  **(n = 298)** | **External validation cohort**  **(n = 80)** | ***P* value** |
| --- | --- | --- | --- |
| **Age, years** | 54.5 (15-81) | 49 (16-82) | **< 0.001** |
| < 60 | 189 (63.4%) | 69 (86.3%) |  |
| ≥ 60 | 109 (36.6%) | 11 (13.8%) |  |
| **Gender (male/female)** | 145/153 | 32/48 | 0.079 |
| **BM blasts, %** | 58.5 (3.5-97) | 62 (17.5-91.2) | 0.947 |
| **WBC, 10^9^/L** | 14.3 (0.4-440) | 13.12 (0.6-239.41) | 0.824 |
| **HB, g/L** | 75 (32-138) | 85 (48-135) | **0.023** |
| **PLT, 10^9^/L** | 40 (3-485) | 42.5 (7-259) | 0.642 |
| **FAB-classification** |  |  | **< 0.001** |
| M0 | 2 (0.8%) | 0 |  |
| M1 | 17 (7.1%) | 6 (9.2%) |  |
| M2 | 117 (48.5%) | 23 (35.4%) |  |
| M4 | 38 (15.8%) | 15 (23.1%) |  |
| M5 | 66 (27.4%) | 21 (32.3%) |  |
| M6 | 1 (0.4%) | 0 |  |
| Not available | 57 (19.1%) | 15 (18.8%) |  |
| **Origin of disease** |  |  | 0.775 |
| De novo AML | 283 (95%) | 77 (96.3%) |  |
| S-AML | 15 (5%) | 3 (3.8%) |  |
| **Treatment** |  |  |  |
| + Venetoclax | 67 (19.1%) | 3 (3.8%) | **< 0.001** |
| + HSCT | 66 (22.1%) | 52 (65%) | **< 0.001** |
| **The n-2023 CN** |  |  | **0.008** |
| Favorable | 90 (30.2%) | 39 (48.8%) |  |
| Intermediate | 93 (31.2%) | 21 (26.3%) |  |
| Adverse | 115 (38.6%) | 20 (25%) |  |
| **The new prognostic model** |  |  | **< 0.001** |
| Favorable | 106 (35.6%) | 49 (61.3%) |  |
| Intermediate | 136 (45.6%) | 27 (33.8%) |  |
| Adverse | 56 (18.8%) | 4 (5%) |  |

**Abbreviations**: BM, bone marrow; WBC, white blood cell; HB, hemoglobin; PLT, platelet; FAB, French-American-British; S-AML, secondary AML. Note: Percentages for FAB classification (M0-M6) are calculated based on the number of patients with available FAB classification data, excluding patients with not available status.
